# Music and awe: complex emotions evoked by tonal dissonance

**DOI:** 10.3389/fpsyg.2026.1767623

**Published:** 2026-04-17

**Authors:** Sara Wilson, Richard F. Braaten, Seth J. Coluzzi

**Affiliations:** 1 Department of Psychological and Brain Sciences, Colgate University, Hamilton, NY, United States; 2 Cognitive Psychology in Context (M.S.), Peabody College, Vanderbilt University, Nashville, TN, United States; 3 Department of Otolaryngology Head and Neck Surgery, Vanderbilt University Medical Center, Nashville, TN, United States; 4 Department of Music, Colgate University, Hamilton, NY, United States

**Keywords:** awe, consonance, dissonance, music, music perception, musicality, psychology of music, tonality

## Abstract

Although previous literature has established a strong connection between awe and music, the influence of consonance and dissonance in this relationship remains largely unexplored. In the present study, participants (*N* = 50) rated consonant and dissonant musical excerpts on their experiences of pleasure, power, and awe. The results indicate that, while consonance tends to evoke pleasure, dissonance elicits stronger feelings of power and awe, up to a certain point. However, when dissonance becomes too pronounced, such as in a highly chromatic or atonal context, feelings of both pleasure and power decline. More dissonant music also significantly predicted increasing feelings of the following specific awe dimensions: *altered time perception, self-diminishment, perceived vastness, physical sensations*, and *need for accommodation*. Musical experience was a significant predictor of the difference in ratings of consonant and dissonant music, with more musically experienced participants deriving greater pleasure, power, and awe, and more *connectedness* and *physical sensations* associated with awe, when listening to increasingly dissonant music. These findings reveal an incongruence between positive affect and emotional intensity, challenging the prevailing assumption in psychological literature that dissonance is merely unpleasant.

## Music and awe: complex emotions evoked by tonal dissonance

Music derives much of its expressive power from the tension and release created by different harmonic relationships. The interplay between consonance and dissonance contributes to this dynamic by introducing contrast and a sense of movement. Consonant harmonies are generally perceived as smooth, harmonious, and stable, whereas dissonant harmonies tend to evoke tension, roughness, and an expectation that the music will eventually resolve. Harmonic dissonance in music is achieved by employing simple vertical intervallic dissonances (e.g., dyads such as seconds, sevenths, tritones, and diminished or augmented intervals), more complex chord structures—i.e., chords that include pitches other than, or in addition to, the root, third, and fifth of a basic major or minor triad—or tone clusters (stacked whole tones and/or semitones).

The perception of and response to harmonic complexity may interact with individual differences in listeners' personalities and musical backgrounds. For example, individuals higher in the personality domain of openness to experience show a preference for chords of greater complexity, with musical experience and engagement mediating this relationship (Nusbaum and Silvia, 2011). Openness to experience is also strongly correlated with profound aesthetic experiences associated with awe—often described as a sense of wonder, amazement, fascination, or being moved and touched (Silvia et al., 2015). This complex emotion of awe has received less attention in psychological and neuroscientific research than milder states like pleasure, liking, and interest. Who tends to experience these powerful states, and what stimuli might serve as catalysts for awe?

In the present study, we investigate whether people exhibiting higher levels of musical engagement and the personality domain of openness to experience are more likely to experience awe. Awe, as conceptualized by Keltner and Haidt (2003), is a complex emotion that includes a sense of self-diminishment in the face of vastness, and a need for accommodation to reconcile one's relationship to an awe-inspiring experience. Keltner and Haidt's conceptualization of awe originates in the discrete emotional tradition (Ekman, 1992). This conceptualization is under debate (Katz and Franz, 2026), with some scholars regarding awe as a combination of other emotions (Konečni, 2005; Halstead and Halstead, 2004), while others question whether it is an emotion at all (Chirico and Gaggioli, 2018). Alternatively, awe could be conceptualized within the framework of the circumplex theory of emotion (Russell, 1980; Wade-Bohleber et al., 2020), presumably as a state of high arousal and either highly positive or negative valence. In the present study, we operationalize awe using the *AWE-S* survey (Yaden et al., 2019), which follows Keltner and Haidt by assessing *vastness* and *need for accommodation*, while also considering *self-diminishment, perception of time, physical sensations*, and *connectedness*.

We ask whether these experiences differ in the presence of music that varies in dissonance. It must be noted that the term “dissonance” is conceptually complex and not uniformly defined. Historically, the classification of certain intervals as consonant or dissonant has shifted within various cultures and eras, reflecting changing aesthetic conventions. For example, major and minor thirds and sixths became consonant only over time in the framework of Western music (Lahdelma and Eerola, 2020). More broadly, scholars disagree about what acoustic or experiential qualities actually constitute dissonance, with explanations ranging from physical properties such as roughness or harmonicity to cultural familiarity and exposure. As a result, consonance and dissonance are often equated with perceptual descriptors, including extent of *pleasantness, tension, smoothness, preference*, etc. In the present study, we adopt a framework grounded in music theory *and* emotional perception to define consonance and dissonance, while acknowledging the limitations and provisional nature of these definitions.

We propose that more complex, dissonant sonorities can facilitate powerful experiences of awe. Dissonance has often been equated with the adjective “unpleasing,” inducing conflicting feelings and strong physiological responses (Dellacherie et al., 2011). However, previous research also indicates that musicians in particular feel attracted to and moved by dissonance, despite the perceived emotion of unpleasantness (Kawakami et al., 2013). To address this paradox, we postulate that dissonant music can serve as a catalyst for the complex emotion of awe, transcending mere pleasure. If this is so, then it would be a mistake to analyze the dissonance/consonance distinction strictly in terms of a displeasure/pleasure framework.

### Aesthetic awe and the neuropsychology of music-evoked emotion

Music is consistently credited across time and cultures as producing sublime moments that feel deeply moving or perspective-shifting, beyond just affection. Referring to his cross-cultural study across 26 cultures, Dacher Keltner writes that participants frequently reported music bringing them feelings of *clarity, epiphany, truth*, and *really knowing their place in the great scheme of life* (Keltner, 2024). Importantly, awe is not solely a pleasant, uplifting emotion; it is often overwhelming, disorienting, and even unsettling. The present study extends insights explored by Hur et al. (2024), who advance a theory of sublime experience that explicitly incorporates confusion, fear, and even pain as central components rather than peripheral byproducts. In this view, awe emerges when existing cognitive frameworks prove insufficient—when one confronts something as vast, complex, or powerful enough to strain the limits of comprehension. Shiota (2021) suggests that awe can be understood as the moment we become aware of a blank space in our mental model of the world, or are confronted with a gap in understanding so immense that it may never be fully resolved. This confrontation can feel like a small earthquake in the mind: destabilizing, cognitively taxing, and emotionally intense. By foregrounding awe's challenging and even painful dimensions, this framework stresses that its generative potential is inseparable from its capacity to unsettle.

Experiences of awe often involve bodily markers such as chills and goosebumps (Yaden et al., 2019), which are believed to index shifts in information processing and attentional focus (Chabin et al., 2020). Individuals who report frequent chills display increased structural connectivity between auditory and emotional regions of the brain, suggesting a more efficient affective-auditory integration (Sachs et al., 2016). Individual differences in the propensity to experience these physiological responses have been linked to trait-level openness to experience (Nusbaum and Silvia, 2011). This Big Five trait, associated with aesthetic sensitivity, imagination, and emotional depth, consistently predicts the frequency and intensity of music-induced chills. Silvia et al. (2015) found that openness—not extraversion or other personality traits—most strongly predicted awe-like responses to music.

Koelsch et al. (2006) further demonstrated that music-induced emotion processing varies over time, with dissonant music producing increased activation in the amygdala, hippocampus, and temporal poles—regions implicated in the processing of negative or complex affect. This temporal unfolding of emotional experience invites further inquiry into how structural features of music shape phenomenological responses, especially in relation to awe. Yet, equating dissonance with “unpleasantness,” as Koelsch et al. (2006) do, may oversimplify the affective power of dissonance. For many listeners, dissonant structures evoke depth, introspection, and poignancy rather than strict mental discomfort or physical auditory roughness (Hur et al., 2024).

### Consonance, dissonance, and the psychology of musical motion

Tonal harmony, a foundational principle of Western music theory, is grounded in the dynamic interplay between consonance and dissonance (Palisca and Moore, 2001). These categories are not binary but exist along a continuum defined by the acoustic complexity of pitch relations. Consonance, characterized by simple frequency ratios between pitches (e.g., the octave at 2:1), is generally perceived as stable, open, and resolved. Dissonance, involving more complex ratios and pronounced auditory roughness, or beating (as explored notably by von Helmholtz, 1863), introduces tension, conflict, and a felt demand for forward movement. The clashing pitches “want” to resolve to a more consonant interval, which drives the experience of anticipation, or “drama,” in music. The emotional and cognitive effects of dissonance are well documented. Bharucha (1984) found that listeners derive greater pleasure from melodies that contain unstable tones when those tones resolve, highlighting the psychological satisfaction of tension-release structures. This suggests that musical enjoyment is linked to predictive coding mechanisms and affective appraisal processes.

Preference for more consonant sounds seems to start at very early developmental stages with newborns opting to listen to consonant rather than dissonant intervals, and a link has been made between the less robust neural responses to dissonant relative to consonant chords that are found at the brainstem level (Mencke et al., 2019). Research suggests that the preference for consonant chords stems from its harmonicity—consonant dyads have many, or more prominent, frequency components in common. Moreover, their partials contain smaller integer ratios than those of dissonant dyads (Mencke et al., 2019). In dissonant intervals, the harmonics are dissimilar and the frequency components lie in the critical bandwidth of the cochlea. For the listener, this leads to the perception of auditory roughness, which underlies sensory unpleasantness.

In Western music, composers such as Haydn, Mozart, Beethoven, and Wagner masterfully leveraged dissonance to generate dramatic impetus, guiding listeners through evolving emotional landscapes (Rosen, 1997). Contemporary reviews of Beethoven's symphonies, for example, describe that the music “sets in motion the machinery of awe, of fear, of terror, of pain, and awakens…infinite yearning” (Hoffmann, 1810), while others in 1805 complained that, with its unusual dissonances, “his music could soon reach the point where one would derive no pleasure from it, unless well trained in the rules and difficulties of the art” (Weiss and Taruskin, 1984). Late nineteenth- and early twentieth-century composers like Wagner and Strauss regularly employed dissonance to manipulate conventional common-practice chords to behave anachronistically (Hutchinson, 2022). In this intentional deviation from expected tonal behavior, the composers exploit a subtle tension between those phenomenological expectations and the unconventional resolutions that arrive in the music.

### Engagement with complex stimuli and the interaction of musical experience

Harmony, the phenomenon of combining notes vertically (i.e., simultaneously) to produce an effect greater than the sum of its parts, is both aesthetic and mathematical in nature (Chan et al., 2019). Why is the pleasing effect of harmony often perceived as exceeding the sum of its individual tones? The inverted-U hypothesis in music proposes that this pleasing effect might depend on musical experience. Witek et al. (2023) tested whether liking ratings for individual chords form an inverted-U relationship with harmonic complexity and whether this pattern differs between musicians and non-musicians. Their findings reveal that non-musicians display peak liking for low harmonic complexity, whereas musicians tend to prefer chords with higher harmonic complexity. Moreover, musicians who favored more complex chords reported higher levels of both active musical engagement and openness to experience, suggesting that musical training and personality jointly shape this preference response.

Parallel insights in artistic complexity come from visual research: Sun and Firestone (2021) found that visual complexity evokes “perceptual curiosity”—a form of attentional engagement prompted by the intricate nature of an image. The present study extrapolates this finding to the auditory realm. In particular, dissonant harmonies, being more acoustically and cognitively complex than consonant ones, may trigger similar curiosity-driven engagement. At the more extreme end, highly dissonant atonal music lacks the tonal center and pitch hierarchy that define tonal music, which has significant cognitive implications. The listener's brain must form novel predictions without clear auditory anchors, giving rise to high “predictive uncertainty” (Mencke et al., 2019).

This characterization resonates with the early work of Leonard Meyer, who first applied information theory to music cognition by proposing that musical styles are internally represented as probabilistic networks shaped through learning and exposure (Meyer, 1957). Contemporary predictive processing scholars extend Meyer's insight by framing music perception as a dynamic, multilevel process in which listeners continuously generate and update probabilistic expectations about *what* will occur and *when* it will occur (Rohrmeier and Koelsch, 2012). Within this framework, expectancy is not limited to local event-to-event transitions, but reflects hierarchical predictions operating across melodic, harmonic, rhythmic, and formal timescales.

Neurophysiological evidence supports this view. Maess et al. (2001) demonstrated using magnetoencephalography (MEG) that violations of musical syntax recruit brain regions implicated in linguistic syntactic processing, including Broca's area and its right-hemisphere homolog. The early right anterior negativity (ERAN), a music-specific event-related potential (ERP), indexes the violation of probabilistic expectations derived from learned tonal regularities. Importantly, recent predictive information-processing models interpret ERAN amplitude not merely as a marker of single rule violation, but as reflecting the precision-weighted prediction error generated when incoming musical events diverge from expected probability *distributions* (Rohrmeier and Koelsch, 2012). Together, these findings support the view that music is processed as a structured, expectation-driven system in which affective and aesthetic responses emerge from the *continuous* modulation of prediction, uncertainty, and surprise.

ERAN amplitude is enhanced by musical training, indicating that experience shapes our predictive musical models (Maess et al., 2001). Musical ability has also been shown to correlate with broader cognitive skills. For instance, Slevc et al. (2016) found that individuals with higher musical ability performed better on both auditory and visual updating tasks, suggesting that the cognitive advantages of musicianship extend beyond domain-specific skills. On an even broader level, Perlovsky et al. (2012) propose that music enables the resolution of cognitive dissonances and supports the accumulation of knowledge across cultures, framing music as a fundamental cognitive function with evolutionary implications.

### Power of dissonance

Kawakami et al. (2013) draw an important distinction between *perceived* and *felt* emotion in response to music. They found that musically trained participants rated dissonant musical passages as expressing negative emotions, yet these same participants reported feeling less unpleasant or even positively moved by the music. This finding helps explain why people often enjoy music that is ostensibly sad or disturbing. In contrast, Dellacherie et al. (2011) found that dissonance evoked stronger physiological arousal and more unpleasant feelings in participants with high musical expertise. These contrasting results may stem from inconsistencies in how affective terms like “unpleasant” are operationalized.

Equating dissonance with unpleasantness is an oversimplification that fails to account for the rich complexity of the emotional responses it can evoke. In fact, dissonance's unpredictability and ambiguity may be precisely what renders it pleasurable to certain listeners. As Mencke et al. (2019) argue, the uncertainty inherent in atonal and dissonant music invites novel cognitive and emotional responses. These styles challenge our expectations and reward us for effortful engagement, especially in musical contexts that prize innovation and novelty. Curiosity, openness, and intellectual engagement are commonly cited among both performers and listeners as necessary conditions for appreciating such music (Mencke et al., 2019). Despite this, neuroscientific studies have rarely treated dissonance as a legitimate style in its own right. Instead, dissonant music is often used as a generic unpleasant stimulus or as a stand-in for fear-inducing or negative affective states.

The prominent theorist Zarlino writes about this very thing in 1558: “for greater beauty and charm dissonances are used, incidentally and secondarily. Although these dissonances are not pleasing in isolation, when they are properly placed… the ear not only endures them but derives great pleasure and delight from them.” And later: “after the ear is offended by a dissonance for a short time, the consonance following it becomes all the more sweet and pleasant” (Zarlino, 1558). The manipulation of tension and release, dissonance and consonance, is among the most fundamental tools in a composer's arsenal. Even for listeners who prefer consonance, it is worth recognizing that the evocative potential of music is inextricably tied to dissonance's complex and often misunderstood power.

### The present study

Musical experience predicts both a higher preference for dissonance (Kawakami et al., 2013) and a physical aversion to dissonance (Dellacherie et al., 2011). We argue that this paradox is the result of too narrow a framework in the academic discourse on the range of emotions evoked by tonal dissonance. The current study addresses this gap in the literature by measuring perceived *power* and *awe* in addition to *pleasure*. We measure power and pleasure using simple Likert scales after each individual musical excerpt, while awe was measured in reference to larger groups of pieces. This approach enabled brief, stimulus-level assessment after each excerpt, which would not be feasible using the comprehensive *AWE-S* administered only at the level of an entire listening sequence. For purposes of scope, this study centers on perceptions of dissonance specific to the Western tonal tradition and within the equal temperament tuning system, and accordingly on participants enculturated within Western musical culture, encompassing both popular and “art” (classical) music.

Although “being moved,” “awe,” and “power” are often used interchangeably in everyday language, empirical work distinguishes these related affective experiences. In particular, “being moved” is characterized as a high-intensity emotional state involving coactivation of positive and negative affect that is not reducible to pleasure alone (Menninghaus et al., 2015). Awe, by contrast, is typically associated with perceived vastness and cognitive accommodation and is commonly assessed using multidimensional instruments designed for extended experiences. In the present work, we use “power” as an umbrella construct to capture participants' immediate sense of being emotionally moved by individual musical excerpts, including experiences marked by sublimity or negatively valenced affect. Consistent with Hur et al. (2024), such powerful experiences may be sublime without being beautiful, overlapping with awe while remaining conceptually distinct.

In the present study, the potential effects of musical experience and openness to experience are investigated, hypothesizing that participants who are more musical and open will be more susceptible to feeling power and awe from dissonance. The potential correlation between musical experience and openness to experience is also of interest (Silvia et al., 2015). The main hypothesis is that consonance will yield more pleasure, while dissonance will yield more power and awe. This is because, while consonance displays a sort of harmonic consistency conducive to the experience of pleasure, dissonance is more complex and thus more difficult to comprehend—qualities characteristic of catalysts for awe.

## Method

### Participants

Fifty participants participated in this study (*N* = 50): 25 males, 23 females, one non-binary participant, and one gender-expansive participant. Thirty-eight of the participants identified as White, four participants identified as both White and Asian, six participants identified as Asian, one participant identified as both White and Hispanic, and one participant identified as African-American. Forty-six participants ranged from ages 18–24, one participant ranged from ages 35–44, one participant ranged from ages 45–54, one participant ranged from ages 55–64, and one participant was age 65+. Participants were recruited from students at university psychology and music courses, as well as musicians at a high school and local choral society.

### Materials

#### Playlists

The pieces were chosen based on their levels of dissonance according to the type, frequency of occurrence, duration, and metrical placement of dissonances to provide a full distribution of samples across the spectrum of consonance to dissonance. Beyond these objective features of consonance and dissonance levels, the pieces in each playlist contain clear qualitative differences. Within the consonant playlist, all excerpts display a prevailing harmonic congruity and stability. They seldom venture far from their pitch (tonal) center and diatonic harmonies or introduce prolonged clashing (dissonant) harmonies, consisting of tonally normative, resolving chord progressions that validate expectancy biases. By contrast, within the dissonant playlist, each excerpt conveys comparatively greater levels of harmonic tension and disagreement. While some pieces resolve these tensions in manners consistent with tonal conventions, others challenge tonal expectations or lack a tonal center altogether, resulting in an ambiguous and unstable musical context. While music categorization is somewhat subjective, a roughly even distribution of pieces by genre was sought, so that about half of the musical excerpts on each playlist qualify as more mainstream rock, while the other half qualify as classical or choral. No excerpts were duplicated between the playlists. Included in the Appendix is a list of the titles and times of the selected excerpts on each playlist.

##### Dissonance levels of musical excerpts

The dissonance levels of the musical excerpts were evaluated based on the type, frequency, duration, and prominence (metrical placement) of the dissonances, resulting in the assignment of a dissonance rating on a 1–5 scale. This analysis focused primarily on dissonances that were harmonic (vertical or simultaneous) rather than melodic (linear or successive) or structural—for example, a chord that is consonant in itself yet that “yearns” toward resolution to another chord, as in the case of a dominant pulling toward the tonic. These criteria, therefore, prioritized simultaneous, clashing sonorities—i.e., those associated with auditory roughness—as measures of dissonance over those that required more long-range hearing, except in instances where the prolonged tensions of structural dissonance played a more salient role (as in the Wagner and Barber examples).

The degrees of the 1–5 scale represent roughly 20% increments of dissonance levels, from 0%−20% for a 1, to 80%−100% for a 5. The only two excerpts to reach the levels of a 5 rating were R. Murray Schafer's atonal *Epitaph for Moonlight* and Béla Bartók's highly chromatic and non-functionally tonal opening movement to the *Music for Strings, Percussion, and Celeste*. The basic features of these ratings are as follows:

**1. Minimal Dissonance**. Triadic harmonies plus occasional dominant-seventh chords with only transient dissonances (neighbor and passing tones less than a beat in duration) in the melody and accompaniment.

**2. Mild Dissonance**. Primarily triadic harmonies with occasional and more prolonged dissonances in the melody (e.g., accented neighbors and suspensions) and accompaniment, including seventh chords beyond dominant sevenths and add6 sonorities.

**3. Moderate Dissonance**. Frequent seventh, ninth, and other added-tone harmonies, including dominants and applied dominants in inversion, along with frequent conventional dissonances (M9, m7, suspensions, appoggiaturas) in the melody. In two of the excerpts—Bach, *Orchestra Suite No. 1 in B minor*; Pink Floyd, “Shine On You Crazy Diamond (Pts. 6–9)” —these dissonances included chord changes over pedal tones (sustained pitches in the bass).

**4. Notable Dissonance**. Prevailing, often consecutive, harmonic dissonance longer than a beat in duration, including in an extended tonal or chromatic context, but still in a fundamentally chordal idiom and with dissonances often unresolved. At times, these dissonances are enhanced by unstable inversions of added-tone harmonies (Beach House, “Days of Candy”), chord changes and dissonances above pedal tones (Led Zeppelin, “No Quarter” and Wagner, *Tristan and Isolde*), and clashes between overlapping harmonies and melody through use of an ostinato (Radiohead, “Weird Fishes / Arpeggi”).

**5. Acute Dissonance**. Overwhelmingly dissonant, including non-chordal harmonies, without regard for resolution, including in an atonal context. The two excerpts that reached this level of dissonance included chord clusters (stacked semitones in Murray Schafer's *Epitaph for Moonlight*) and chromatic and non-triadic harmonies consisting of semitones, tritones, and stacked wholetones (Bartók's *Music for Strings, Percussion, and Celeste*).

##### Independent validation of dissonance levels of musical excerpts

To independently validate the dissonance level of the musical excerpts, three independent raters listened to each excerpt and rated them on a 1–9 scale ranging from 1 (“entirely consonant”) to 9 (“entirely dissonant”). The purpose of this independent validation was not to provide a detailed analysis of the level of dissonance, but simply to validate that the musical excerpts that we presented to participants in the block of consonant excerpts were indeed perceived by independent raters as less dissonant than the excerpts that we presented in the dissonant block. The raters were trained orchestral musicians who had studied music theory, and no particular definition of consonance and dissonance was given to them as they had a good musical understanding of these concepts. Two of the raters were professional orchestra wind players, and the third was a college orchestral string player. The order of the presentation of the excerpts for ratings was randomized so that excerpts designated as consonant and dissonant by the authors were intermixed. The independent raters did not know whether the excerpts had been classified as consonant or dissonant and had no knowledge of the ratings of participants.

Mean dissonance ratings for the three raters was 2.97 for consonant excerpts and 6.37 for dissonant excerpts. A Mann-Whitney U-test found this difference to be statistically significant, *U* = 2.50, *p* = 0.002. The range of the mean ratings was 2.0–5.0 for the consonant excerpts, and 3.67–8.0 for the dissonant excerpts. Mean interrater correlation was *r* = 0.613. All dissonant excerpts were rated as more dissonant than all consonant excerpts with one exception: the excerpt of *O Magnum Mysterium* by Lauridsen was given a mean rating of 3.67, which was at the higher end of the range given to excerpts designated by the authors as consonant.

#### The big five inventory (BFI)

The *Big Five* structure provides a replicable representation of the major dimensions of trait description in English, namely: *openness to experience, conscientiousness, extraversion, agreeableness*, and *neuroticism* (Goldberg, 1992). The five-factor structure seems to generalize reliably across different types of samples, raters, and methodological variations when comprehensive sets of variables are factored. In the present study, we only analyzed the trait of openness to experience, taking the mean of the inventory items associated with this specific trait.

#### Scale of musical experience (SME)

To assess musical experience, scores on 10 questions from four of the five categories of the SME (categories 1–4) were analyzed (Goncy and Waehler, 2006). These original 40 statements were designed to measure five categories applicable to musical experience: (1) *knowledge and experience in different genres of music*; (2) *improvisation, composition and theory*; (3) *performance aspects*; (4) *self-awareness as a musician*; (5) *musical interest and external application*. The questions we chose are the following:

#8: *I use a wide range of dynamics in performance*. (category #3)

#12: *I understand musical notation very well*. (category #2)

#13: *Others would rate me as above average on my instrument/voice*. (category #3)

#17: *I frequently compose musical pieces*. (category #2)

#19: *I feel that I am an excellent musician*. (category #4)

#23: *When performing or singing a piece, I pay attention to marked dynamics*. (category #3)

#28: *My musical skill level can be considered at a college-level ability*. (category #3)

#36: *I would feel comfortable performing musical pieces by myself*. (category #1)

#37: *I am very good at music theory*. (category #2)

#38: *I am a good sight-reader*. (category #3)

In our shortened version of this scale, we decided to omit questions from category five, therefore excluding responses to questions directed toward people who are interested in music but may lack practical musical knowledge or skill. This is because we wanted our variable of “musicality” to be more representative of constructs such as competence and expertise rather than interest. From there, we chose questions from the remaining categories that, in our opinion, most explicitly spoke to competence and expertise. This included dynamics, performance, composition, theory, and metacognition of musical skill. The mean responses to these 10 questions was taken as the measure of musical experience.

#### Awe experience scale (AWE-S)

The *AWE-S* is a stable and reliable 6-factor state measure of the complex emotion of awe (Yaden et al., 2019). This scale synthesizes previous research on awe and opens up new and informative research possibilities for exploring various aspects of awe. These six dimensions of awe include: *altered time perception* (F1); *self-diminishment* (F2); *connectedness* (F3); *perceived vastness* (F4); *physical sensations* (F5); *need for accommodation* (F6). In the present study, use of this multifactorial scale has made it possible to distinguish the different roles that each dimension of awe plays on various subsequent outcomes.

### Procedure

This experiment was conducted entirely in-person with each individual participant. All of the participants read and signed the *Certificate of Informed Consent* before beginning the experimental task. Participants first answered demographic questions and completed the *Big Five Inventory Questionnaire* (*BFI*). Participants then completed the *Scale of Musical Experience* (*SME*). They then listened to two different playlists: one consisting of 10 consonant musical excerpts (dissonance levels 1–3), and one containing 10 dissonant excerpts (dissonance levels 3–5)—with each excerpt lasting 1 min. The order in which the two playlists were presented was counterbalanced among the participants.

During the listening experience, participants were encouraged to be in a relaxed state, however that might look. All participants used *Bose QuietComfort Ultra Headphones* for the listening portion, and the volume was set the same for all participants. All studies were conducted facing a window, and participants could look out the window, lay down, close their eyes, etc. After each excerpt, participants rated how pleasurable and powerful the excerpt was on a 10-point Likert scale (1 being low, and 10 being high). Participants were told explicitly that “pleasure” refers to how much the participants enjoyed the piece more basically (strictly positive affect), while “power” refers to how much the participants were emotionally moved by the piece in any way. When they completed each playlist, they completed the *Awe Experience Scale* (*AWE-S)*. Upon completion of the experiment, participants were verbally debriefed regarding the aim of the study. The duration of the experimental task took approximately 1 h to complete.

## Results

### Effects of consonance and dissonance on pleasure, power, and awe

**Figure 1:**
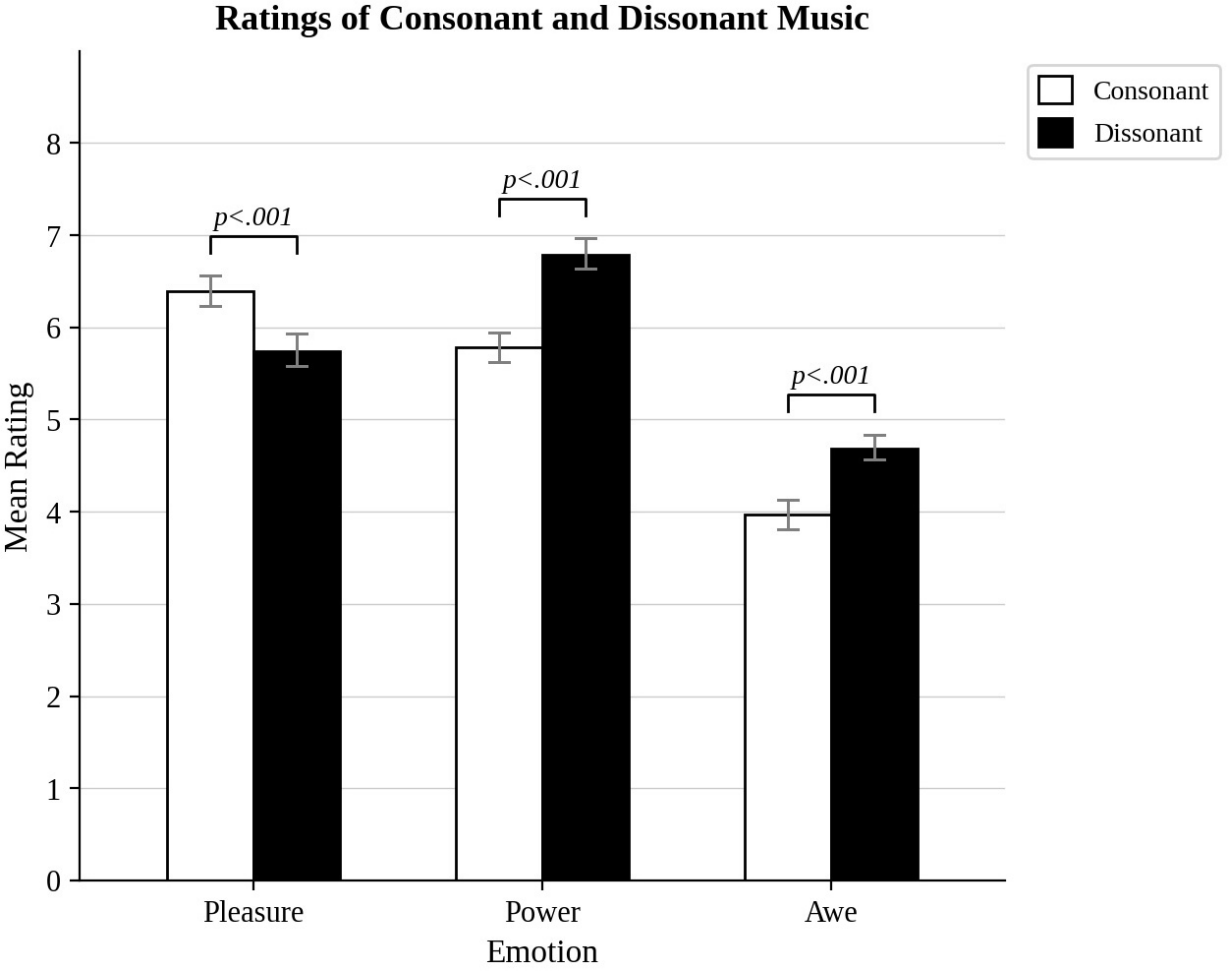
Pleasure, power, and awe ratings of consonant and dissonant music.

The results for pleasure, power, and awe revealed significant differences between the experience of consonance and dissonance (see Figure 1). There was a significant difference in pleasure ratings for consonance (*M* = 6.40) as compared to dissonance (*M* = 5.74), *t*_(49)_ = 3.87, *p* < 0.001, *d* = 0.55. There was a significant difference in power ratings for consonance (*M* = 5.79) as compared to dissonance (*M* = 6.79), *t*_(49)_ = 6.13, *p* < 0.001, *d* = 0.87. There was a significant difference in awe ratings for consonance (*M* = 3.97) as compared to dissonance (*M* = 4.68), *t*_(49)_ = 4.36, *p* < 0.001, *d* = 0.62. Thus, while consonance was more pleasurable, dissonance was more powerful and awesome.

### Non-linear relationship between dissonance and pleasure/power

**Figure 2:**
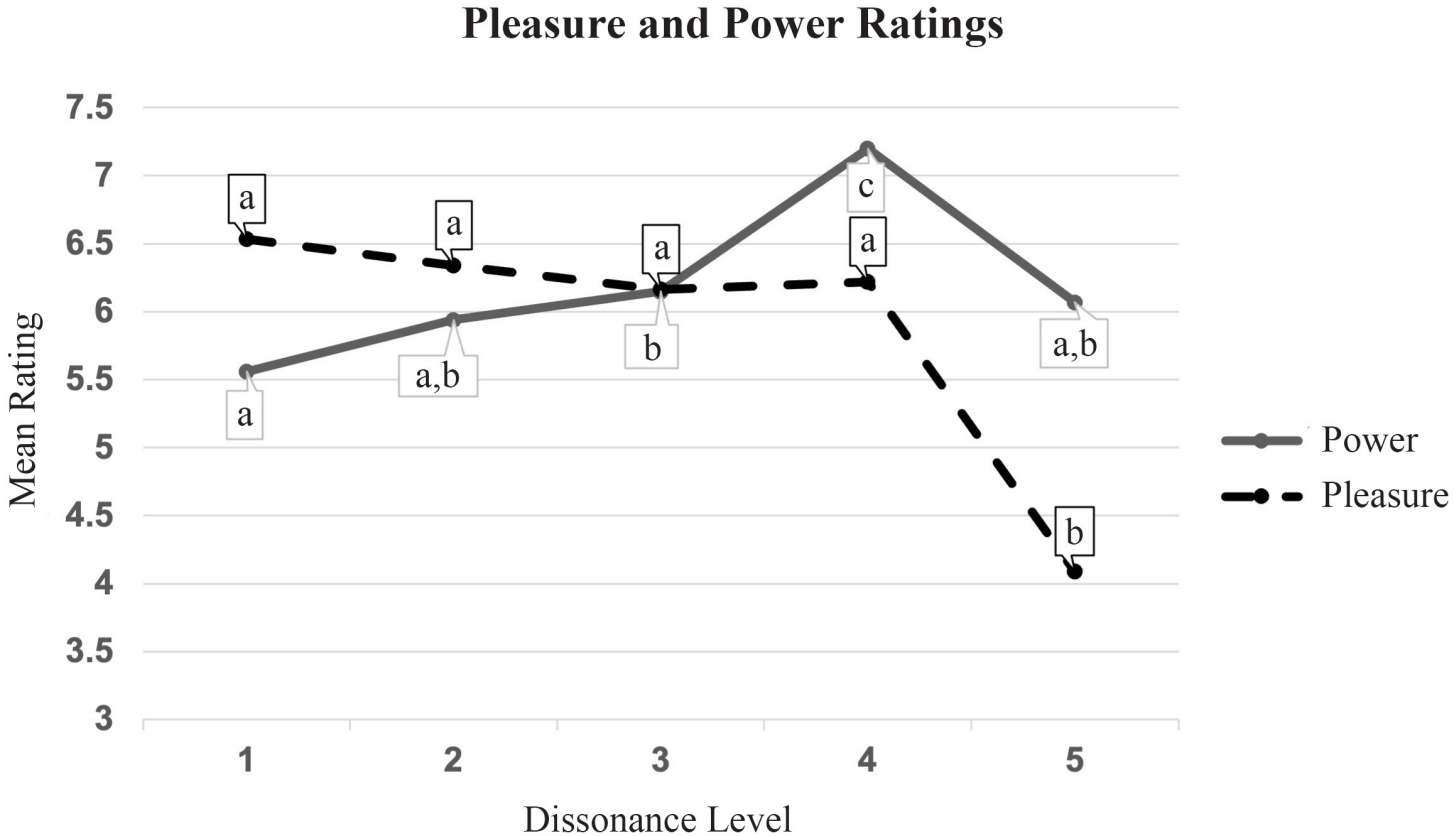
Mean pleasure and power ratings of the 20 excerpts by dissonance (data points within a line with the same letter notation are not significantly different, Tukey's *p* > 0.05).

Ratings of the 20 musical excerpts varied by the five dissonance levels. There was a significant effect of dissonance on pleasure ratings, *F*_(4, 196)_ = 55.50, *p* < 0.001, η^2^ = 0.29. Figure 2 shows the 20 excerpts categorized by dissonance level. As shown in Figure 2, pleasure did not change much with increasing dissonance until there was a sharp dropoff in pleasure at the highest dissonance level. There was also a significant effect of dissonance on power ratings, *F*_(4, 196)_ = 19.1, *p* < 0.001, η^2^ = 0.14, with increasingly dissonant excerpts being gradually experienced as more powerful, except for the highest dissonance level, for which there was a dropoff in power rating. Awe could not be assessed by dissonance level because awe was only rated after each block of 10 excerpts, not after each of the 20 excerpts.

### Effects of musical experience and openness to experience

There was a significant correlation between openness to experience and musical experience, *r*(49) = 0.414, *p* < 0.05. In order to see if either of these variables predicted differences in ratings to the consonant and dissonant music, multiple linear regressions were done on the difference scores between responses to consonant and dissonant excerpts for each of the dependent measures: pleasure, power, and awe, with musical experience and openness to experience as predicting variables. For pleasure, the overall regression model was significant, *F*_(2, 47)_ = 8.54, *p* < 0.001, *R*^2^ = 0.27. Musical experience predicted differences in pleasure responses between consonant and dissonant excerpts (β = 0.41, *p* = 0.004), while openness to experience did not (β = 0.18, *p* = 0.186). For power, the overall regression model was significant, *F*_(2, 47)_ = 12.6, *p* < 0.001, *R*^2^ = 0.35. Musical experience predicted differences in power responses between consonant and dissonant excerpts (β = 0.59, *p* < 0.001), while openness to experience did not (β = 0.01, *p* = 0.959). For awe, the overall regression model was marginally significant, *F*_(2, 47)_ = 2.66, *p* = 0.08, *R*^2^ = 0.10. Neither musical experience (β = 0.21, *p* = 0.169) nor openness to experience (β = 0.17, *p* = 0.279) significantly predicted differences in awe responses between consonant and dissonant excerpts.

**Figure 3:**
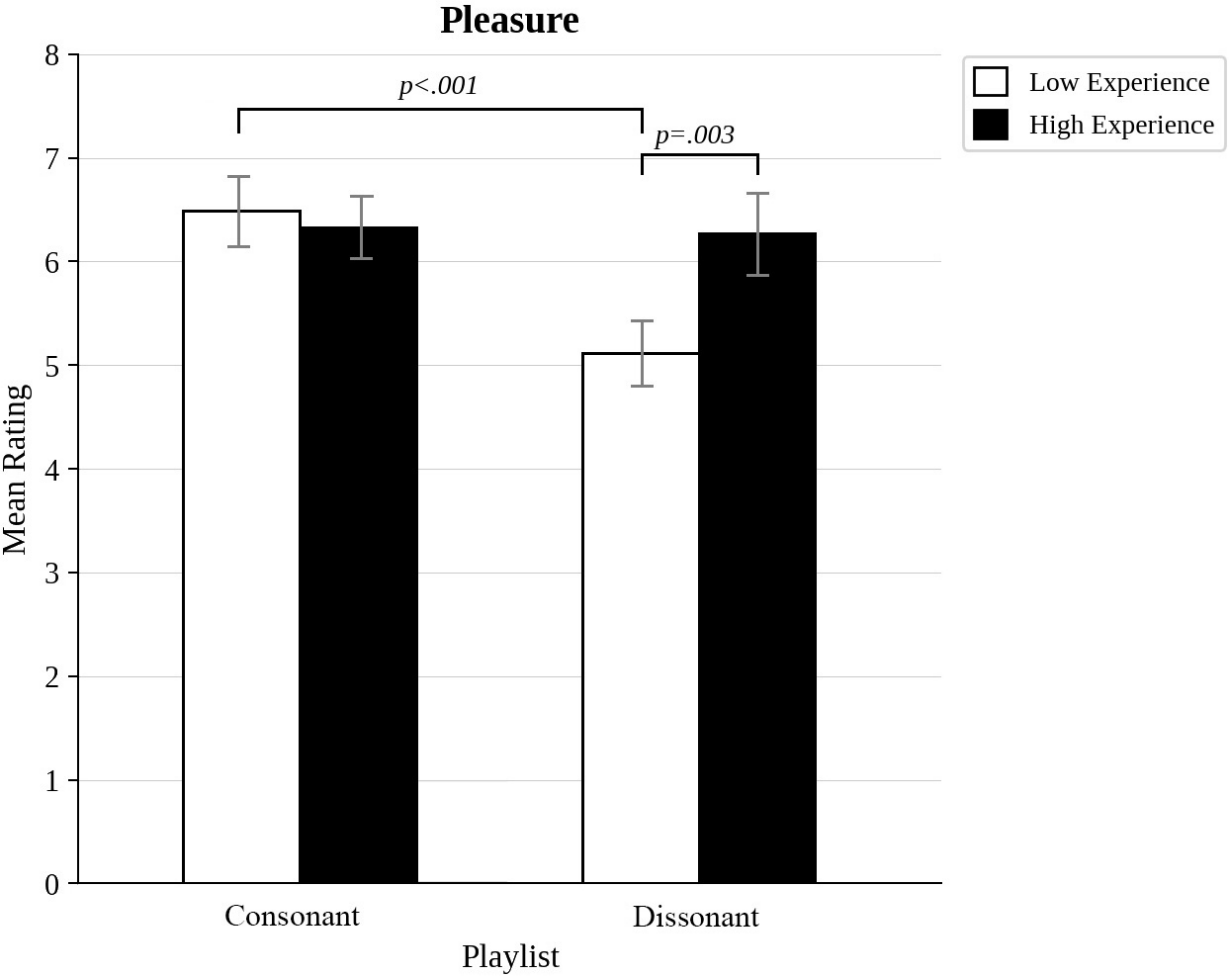
Pleasure ratings for consonant and dissonant musical excerpts for participants with high and low musical experience.

**Figure 4:**
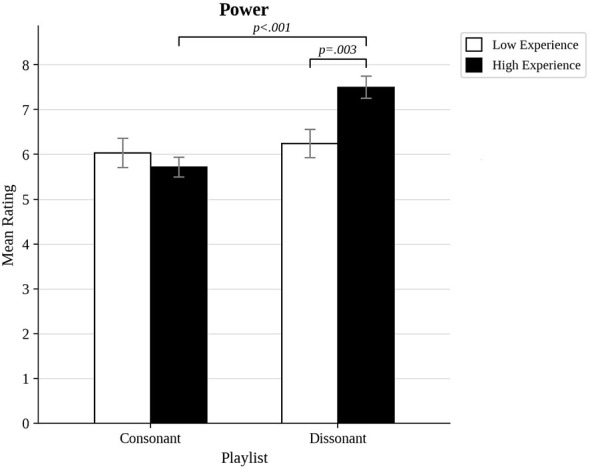
Power ratings for consonant and dissonant musical excerpts for participants with high and low musical experience.

**Figure 5:**
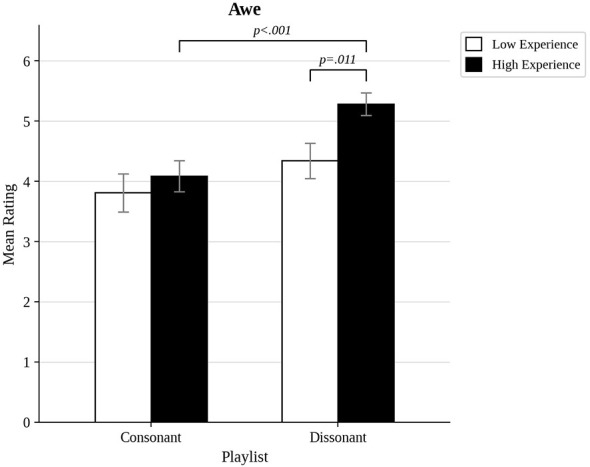
Awe ratings for consonant and dissonant musical blocks for participants with high and low musical experience.

We also compared the most musically experienced participants to the least musically experienced ones, and those most open to experience to those least open to experience, by trichotomizing the distributions of scores to compare approximately the top third to the bottom third (the highest and lowest 17 scores out of 50). As shown in Figure 3, there was a significant interaction between musical experience and dissonance for pleasure ratings, *F*_(1, 32)_ = 9.69, *p* = 0.004, η^2^= 0.05. *Post hoc t*-tests indicated that while there was no effects of experience for consonant excerpts (*p* = 0.558), there was a significant difference for dissonant excerpts (*p* = 0.003), because less experienced participants found dissonant excerpts less pleasurable than consonant ones (*p* < 0.001). A similar pattern of results was found for power ratings (see Figure 4). There was a significant interaction between musical experience and dissonance, *F*_(1, 32)_ = 18.7, *p* < 0.001, η^2^= 0.08. High and low experienced participants did not differ in power ratings of consonance (*p* = 0.558), but they did for dissonance (*p* = 0.003), because more experienced participants found dissonance to be more powerful than consonance (*p* < 0.001). A significant interaction between musical experience and dissonance was also found for awe, *F*_(1, 32)_, *p* = 0.023, η^2^= 0.02 (see Figure 5). Once again, this was because the groups did not differ in their responses to consonance (*p* = 0.549), but they did for dissonance (*p* = 0.011) because more experienced participants rated dissonant excerpts higher for awe (*p* < 0.001).

For openness to experience, the results were different. For all ratings, there were significant main effects for dissonance: for pleasure, *F*_(1, 32)_ = 9.92, *p* = 0.004, η^2^= 0.06; for power, *F*_(1, 32)_ = 48.34, *p* < 0.001, η^2^= 0.14; and for awe *F*_(1, 32)_ = 10.88, *p* = 0.002, η^2^= 0.09. Pleasure was rated higher for consonance, and power and awe were rated higher for dissonance. However, there were no significant interactions with openness to experience. Although we did not find significant interactions between dissonance and openness to experience, it should be noted that there was much less variability in the openness to experience scores (*M* = 3.96, *SD* = 0.66) than the musical experience scores (*M* = 3.02, *SD* = 1.96).

### Various dimensions of awe

The *Awe-Experience Scale* represents a comprehensive measure of six critical dimensions of awe, including: (1) *altered time perception*, (2) *self-diminishment*, (3) *connectedness*, (4) *perceived vastness*, (5) *physical sensations*, and (6) *need for accommodation*. Table 1 shows awe ratings for each of the six dimensions for consonant and dissonant blocks of music for the third of participants highest and lowest in musical experience for each of the six dimensions. Table 1 also shows *F* and *p*-*values* from two-way ANOVAs with dissonance as the repeated measures factor (consonant and dissonant blocks) and musical experience (high or low) as the between groups factor. As shown in Table 1, the main effect for dissonance was significant for 5 of the 6 dimensions, with connectedness being marginally significant. Also shown in Table 1, there were significant interactions of musicality with connectedness and physical sensations, and a marginally significant interaction for vastness. *Post hoc t*-tests found that for connectedness, responses for consonance did not differ for low-experienced (*M* = 4.42) and high-experienced participants (*M* = 4.25), *p* = 0.749, but for dissonance, more experienced participants perceived more of a sense of connectedness (*M* = 5.04) than did low-experienced participants (*M* = 4.34), *p* = 0.003. For physical sensations, responses for consonance also did not differ for low-experienced (*M* = 2.71) and high-experienced participants (*M* = 3.38), *p* = 0.205, but for dissonance, more experienced participants perceived more physical sensations (*M* = 4.86) than did low-experienced participants (*M* = 3.06), *p* = 0.001.

**Table 1:** Analyses of responses to consonant and dissonant music on the awe experience scale.

Awe dimension	*Mean awe responses*		*Main effect of dissonance*			*Interaction with musicality*		
	Consonant	Dissonant	*F*	*p*	*η2*	*F*	*p*	*η^2^*
Time	4.61	5.45	**16.02**	**< 0.001**	**0.11**	0.49	0.489	0.03
Self	3.44	4.75	**28.49**	**< 0.001**	**0.17**	0.30	0.586	0.00
Connectedness	4.34	4.69	4.01	0.054	0.01	**6.10**	**0.019**	**0.02**
Vastness	4.63	5.64	**25.55**	**< 0.001**	**0.11**	3.92	0.056	0.02
Sensations	3.04	3.96	**17.68**	**< 0.001**	**0.08**	**6.70**	**0.014**	**0.03**
Accommodation	3.49	4.64	**42.70**	**< 0.001**	**0.19**	0.37	0.549	0.00

Bold values highlight statistical significance at the *p* < 0.05 level.

## Discussion

This is the first study to examine the relationship between consonance and dissonance in music and the conscious state of awe. The results indicate that dissonance level is strongly related to the experience of awe. While the consonant excerpts were more pleasurable, the dissonant excerpts were more powerful and elicited stronger feelings of awe. Listeners may have derived pleasure from the predictability, harmoniousness, and coherence of consonant musical passages. In contrast, dissonant excerpts challenge syntactical expectations and confront listeners with uncertainty and complexity, conducive to the experience of awe. The juxtaposition of pleasure and awe highlights the complexity of emotional responses to consonance and dissonance whereby the relationship between positive affect and emotional intensity is non-linear. While displeasure and awe may seem diametrically opposed, we find that they can coexist within the same musical composition.

Musical experience played a critical role in this main effect. As musical experience increased, there was an increasing tendency to take pleasure in dissonance relative to consonance, and to find dissonance more powerful and awesome. Similarly, within the six dimensions of awe, musical experience moderated the relationship between dissonance and feelings of connectedness, and the experience of physical sensations. Participants with more musical experience experienced increased feelings of connectedness and physical sensations for dissonance relative to consonance. Conversely, participants with lower musical experience exhibited less preference for dissonance in terms of pleasure, and perceived an insignificant difference in power and awe between consonance and dissonance. It will be interesting for future research to explore the mechanisms driving this interaction—whether it be structural differences in the brain, genetic bases, mere familiarity, or some combination.

We also considered the effect of openness to experience on responses to consonant and dissonant music. Openness to experience was highly correlated with musical experience, and when the two were entered into regression models, musical experience significantly predicted differences in pleasure, power, and some aspects of awe in response to consonant and dissonant excerpts, but openness to experience did not. Thus, in this study, musical experience was a more significant factor than openness to experience in participants' responses. It should be noted that there was more variability in responses to the musical experience scale than to the openness to experience scale, likely because we sought out participants that varied in musical experience. Participant characteristics, including musical engagement and/or being college students, may have resulted in participants relatively high in openness to experience. It is certainly possible that openness to experience would be a significant predictor of responses to consonant and dissonant music with a more diverse group of participants.

As Figure 2 demonstrates, the correlation between increasing dissonance and increasing levels of perceived power is not direct. Rather, there is a threshold between dissonance-levels 4 and 5 at which listeners begin to report feeling decreased power in the music. This threshold coincides within the boundary between extended tonality (dissonance-level 4)—i.e., music that uses triadic harmonies with added dissonances in both conventional and unconventional ways, but that is still grounded by a tonal (pitch) center—and exceptional chromatic and non-chord-based dissonance extending to the level of atonality (dissonance-level 5)—music that lacks a pitch center altogether, and that uses dissonance freely, without triadic harmonies and conventional tonal syntax. This same dropoff pertains to perceived pleasure as well (see Figure 2), which decreases notably with the most dissonant examples. This “tipping point” in perceived power and pleasure suggests that there is a maximum amount of dissonance in terms of eliciting increased pleasure and power, and that this tipping point may coincide with the relinquishing of a tonal grounding, triadic-based harmonies, and the organizational systems of tonality that distinguish atonal music. We could speculate that humans perhaps crave a kind of “goal-directed” tension that is absent in atonality and other highly dissonant music that is devoid of tonal, chordal-based coherence, although it is a claim that certainly warrants further study.

By separately analyzing all six categories of the *Awe Experience Scale*, we were able to more granularly isolate which dimensions of awe are being implicated in the experience of dissonance. Specifically, there were significant main effects of dissonance observed in *altered time perception, self-diminishment, perceived vastness, physical sensations*, and *need for accommodation*. The observed effect on the *need for accommodation* suggests that experiencing awe, particularly induced by dissonant music in this case, may require individuals to make significant mental adjustments to integrate and process the experience. There was only a marginally significant difference in responses to consonant and dissonant music for feelings of *connectedness*, yet musically experienced participants had significantly greater feelings of connectedness with dissonant music. Recognizing the impact of musical experience on feelings of connectedness could potentially inform therapeutic interventions and music-based interventions for various populations.

Perhaps most interesting was the analysis of *physical sensations*. The significant main effect of dissonance corroborates previous research indicating that dissonant music elicits stronger physiological responses compared to consonant music (Dellacherie et al., 2011). This reaffirms the notion that dissonance, with its inherent tension and unpredictability, triggers physiological arousal, such as chills/goosebumps, increased heart rate, or eye-widening, indicative of heightened emotional engagement. This effect was significantly amplified in participants higher in musical experience. Future research could explore how musical experience and personality traits may modulate physiological responses to music in the context of therapeutic interventions targeting emotional regulation and stress reduction.

There is reason to believe that awe is somewhere in between an emotion and an altered state of consciousness (Yaden et al., 2019). The main effects of dissonant music on the experience of time, sense of self, and vastness are particularly notable. These perceptual changes mark a distinction between awe and other emotions, as alterations to such fundamental faculties of consciousness are unusual in the emotion literature. While emotions typically evoke subjective experiences within a relatively stable framework of consciousness, awe seems to transcend these boundaries, inducing profound alterations to fundamental faculties of perception and cognition. Consequently, the current study represents an intersection of inquiry into complex concepts within both psychology and music theory—the enigmatic nature of awe and the mysterious phenomenology of dissonance. Investigating these two aspects concurrently has the potential to significantly enrich the literature in both disciplines.

### Limitations

It was difficult to control various aspects of the experimental task. Music perception and categorization is inherently subjective, and we tried our best to control for the musical genre across both playlists to mitigate the effects of personal preferences. This included ensuring that the consonant playlist had a relatively even split of pieces in major and minor keys, as well as other elements such as instruments, dynamics, lyricism, etc. Furthermore, both playlists were curated in collaboration with music theory experts to have a professional perspective on how to control for major qualitative differences between the two playlists other than tonality.

Nonetheless, there are certainly other elements of the musical excerpts besides the extent of dissonance or consonance that could have evoked feelings of pleasure, power, and awe, such as tempo, texture, timbre, etc. Although careful attention was given to these qualities in the construction of these playlists, we cannot rule out their potential influence. This might also include familiarity with or personal memories associated with individual song excerpts. It is possible that participants with more musical experience had previously encountered some of the pieces through study or performance, leading to deeper structural anticipation, analytic listening, or autobiographical associations. However, it is unclear why such familiarity would uniformly enhance power or awe over perhaps attenuating them by reducing uncertainty and surprise—two features often implicated in awesome aesthetic experiences.

While the extent of dissonance was operationalized as the primary experimental manipulation, the emotional impact of music is inherently multidimensional. Of course, it would have been more controlled to simply present individual chord progressions. However, this would have made it very difficult to measure awe—a conscious state that requires a rich and multi-dimensional experience. On this note, it might have been difficult to experience genuine awe in a controlled study setting such as this one. Therefore, future research, especially in the context of awe, will have to find novel ways to reconcile internal validity with ecological validity.

Finally, the present sample is skewed toward young, Western participants, which limits generalizability. Especially given the lively cultural disputes over meaning and perception of dissonance, it will be imperative for future research to recruit diverse demographics of participants. Expanding samples to include participants from non-Western musical traditions and more varied age groups would help clarify whether the observed emotional responses reflect universal perceptual mechanisms or culturally learned frameworks. Such diversification is essential for developing a more globally representative account of how dissonance is experienced and understood.

### Implications

The dichotomy between the pleasure derived from consonance and the powerful and awesome resonance of dissonance speaks to the complexity of our emotional responses to music. The fine-grained analysis of awe across different dimensions has allowed us to begin to isolate what experiences within awe are perhaps most salient in musical contexts. Further research could investigate the neural mechanisms underlying these experiences, potentially drawing parallels with other forms of aesthetic or spiritual experiences, such as meditation or psychedelic states. Given the potential of dissonant music in evoking awe and challenging cognitive boundaries, there could be applications for using such music in therapeutic contexts. Future studies could explore the efficacy of dissonant music in facilitating emotional processing, promoting mindfulness and creativity, and even transcendence. The paradoxical nature of finding awe in discomfort and ambiguity raises broader questions about the nature of beauty, meaning-making, and existential exploration. Future interdisciplinary inquiries could integrate philosophical, psychological, and aesthetic perspectives to deepen our understanding of the role of dissonance in human experience and innovation.

## Author's note

Publication of this article was supported by the Faculty Research Council of Colgate University. Portions of these findings were presented as a poster at the 2025 conference of the Society for the Psychology of Aesthetics, Creativity, and the Arts, APA Division 10, New Haven, CT, USA. This research was originally submitted as a thesis to the Department of Psychological & Brain Sciences at Colgate University in partial fulfillment of the requirements for the degree of Bachelor of Arts with High Honors.

## Data Availability

The raw data supporting the conclusions of this article will be made available by the authors, without undue reservation.

## Appendix

### Playlists

Consonant playlist (song titles and times of the selected excerpts):

1. *Here Comes The Sun* – The Beatles: 0–1:00
2. *Misty Morning* – Bob Marley & The Wailers: 0–1:00
3. *Everywhere* – Fleetwood Mac: 0–1:00
4. *Eclipse* – Pink Floyd: 0–1:00
5. *High and Dry* – Radiohead: 0:15–1:15
6. *Goldberg Variations: BWV 988: Aria* – Johann Sebastian Bach: 2:04–3:04
7. *Clair De Lune* – Claude Debussy: 4:06–5:06
8. *Waltz in A Minor, Op. Posth., B. 150* – Frédéric Chopin: 0–1:00
9. *Overture (Suite) No. 2 in B Minor* – Johann Sebastian Bach: 0:30–1:30
10. *Shostakovich: Fugue No. 7 in A Major* – Dmitri Shostakovich: 1:43–2:43

Dissonant playlist (song titles and times of the selected excerpts):

1. *No Quarter* – Led Zeppelin: 0–1:00
2. *Weird Fishes/ Arpeggi* – Radiohead: 3:33–4:33
3. *Shine On You Crazy Diamond (Pts. 6–9)* – Pink Floyd: 0:55–1:55
4. *Days of Candy* – Beach House: 0–1:00
5. *Epitaph for Moonlight* – R. Murray Schafer: 0–1:00
6. *Tristan und Isolde: Prelude und Liebestod* – Richard Wagner: 15:43–16:43
7. *O Magnum Mysterium* – Morten Lauridsen, Chamber Choir of Europe: 4:57–5:57
8. *Music for Strings, Percussion and Celesta, Sz. 106: I. Andante tranquillo* – Béla Bartók: 3:55–4:55
9. *Adagio for Strings, Op. 11* – Samuel Barber: 0–1:00
10. *The Maze* – Anthony Willis, London Contemporary Orchestra: 1:00–2:00
